# Liposomal PHD2 Inhibitors and the Enhanced Efficacy in Stabilizing HIF-1α

**DOI:** 10.3390/nano12010163

**Published:** 2022-01-03

**Authors:** Cheng-Bang Jian, Xu-En Yu, Hua-De Gao, Huai-An Chen, Ren-Hua Jheng, Chong-Yan Chen, Hsien-Ming Lee

**Affiliations:** 1 Institute of Chemistry, Academia Sinica, Taipei 11529, Taiwan; r01223223@ntu.edu.tw (C.-B.J.); allenyu2932@gmail.com (X.-E.Y.); student719@msn.com (H.-D.G.); eam012331@gmail.com (H.-A.C.); renhuajheng@gmail.com (R.-H.J.); andy6111ha@gmail.com (C.-Y.C.); 2 Department of Chemistry, National Taiwan University, Taipei 10617, Taiwan; 3 Nano Science and Technology Program, Taiwan International Graduate Program, Academia Sinica and National Taiwan University, Taipei 11529, Taiwan; 4 Department of Chemistry, National Central University, Taoyuan City 320317, Taiwan

**Keywords:** liposome, remote loading, PHD2 inhibitor, HIF-1, IOX2, vadadustat, roxadustat

## Abstract

Prolyl hydroxylase domain-containing protein 2 (PHD2) inhibition, which stabilizes hypoxia-inducible factor (HIF)-1α and thus triggers adaptation responses to hypoxia in cells, has become an important therapeutic target. Despite the proven high potency, small-molecule PHD2 inhibitors such as IOX2 may require a nanoformulation for favorable biodistribution to reduce off-target toxicity. A liposome formulation for improving the pharmacokinetics of an encapsulated drug while allowing a targeted delivery is a viable option. This study aimed to develop an efficient loading method that can encapsulate IOX2 and other PHD2 inhibitors with similar pharmacophore features in nanosized liposomes. Driven by a transmembrane calcium acetate gradient, a nearly 100% remote loading efficiency of IOX2 into liposomes was achieved with an optimized extraliposomal solution. The electron microscopy imaging revealed that IOX2 formed nanoprecipitates inside the liposome’s interior compartments after loading. For drug efficacy, liposomal IOX2 outperformed the free drug in inducing the HIF-1α levels in cell experiments, especially when using a targeting ligand. This method also enabled two clinically used inhibitors—vadadustat and roxadustat—to be loaded into liposomes with a high encapsulation efficiency, indicating its generality to load other heterocyclic glycinamide PHD2 inhibitors. We believe that the liposome formulation of PHD2 inhibitors, particularly in conjunction with active targeting, would have therapeutic potential for treating more specifically localized disease lesions.

## 1. Introduction

The induction of cellular hypoxia-inducible factor (HIF) levels leading to adaptation responses to reduced oxygen availability (hypoxia) [1], such as reestablished oxygen homeostasis, shifted metabolism, and regulated cell growth, has become a promising strategy for treating several diseases [2,3]. HIF prolyl hydroxylase domain-containing protein (PHD) responsible for downregulating HIF levels [4] is thus a crucial therapeutic target. HIFs are heterodimeric proteins that consist of α subunits and β subunits [5]. In mammals, there are two functional α subunits, ubiquitously expressing HIF-1α and tissue-specific HIF-2α [6]. Under sufficiently oxygenated (normoxic) conditions, both HIFα isomers are downregulated through PHD-mediated hydroxylation at two specific proline residues [7,8], which tags them for polyubiquitination and subsequent proteasomal degradation [9]. The PHD-catalyzed hydroxylation of HIFα requires molecular oxygen and α-ketoglutarate (α-kG, an intermediate in the Krebs cycle) as enzyme substrates; PHDs, therefore, act as oxygen sensors that directly regulate HIFα protein levels as a function of environmental oxygen levels [10]. As a result, HIF α subunits are stabilized under hypoxia, allowing the formation of HIF heterodimeric complexes for the transcriptional regulation of downstream hypoxic-adaptation genes [11].

Among three PHD isomers (PHD1, PHD2, and PHD3) [12], PHD2 is the most critical in mediating HIF-1α protein degradation under normoxia [13], making it an essential target. Many enzyme inhibitors selective against PHD2 over a broad spectrum of α-KG-dependent dioxygenases have, since been, discovered [14,15]. Most of them are α-kG mimetics that can bind to the catalytic site of PHD2 [16]. These PHD2 inhibitors as HIFα stabilizers have received the most clinical success as an alternative treatment of anemia in patients with chronic kidney diseases by stimulating renal and hepatic erythropoietin production [17]. In addition, they have been demonstrated to be protective against ischemia-reperfusion injury in experimental ischemic diseases [18,19,20]. Recently, a growing body of evidence has linked aberrantly high PHD expression to several human cancers [21,22,23] and suggested that inhibiting PHD2 can be a potential therapeutic for cancer [24,25,26]. Moreover, PHD2 inhibition in endothelial cells has been shown to normalize the tumor vasculature through HIFα stabilization [27,28,29] and thus sensitize tumors to chemotherapy, resulting in improved treatment outcomes in xenograft mice [27,30]. Despite the potential applications, the systemic administration of small-molecule PHD2 inhibitors has raised concerns about undesired side effects [31,32] and the potential risks of off-target biodistribution [33,34]. For example, PHD2 inhibitor vadadustat failed to meet the criterion for cardiovascular safety in recent clinical trials [35], eventually leading to its U.S. FDA rejection for use. This concern should be taken into account, in particular when dealing with localized disease lesions. In addition, the efficacy of acidic PHD2 inhibitors may be restricted by their poor tissue distribution when entering the systemic circulation due to high plasma protein binding [36].

Drug nanoformulation is a viable option for improving the on-target tissue accumulation of drugs [37,38]. Recently, a relevant study demonstrated that, in a mouse model of acute kidney injury, the intravenous injection of polyamidoamine dendrimer-wrapped PHD2 siRNA nanoparticles with folate decoration results in nanoparticle accumulation most in the kidneys and significantly reduces reperfusion injury [39]. To date, however, the nanoformulation of PHD inhibitors remains largely unexplored. Liposome, a clinically proven therapeutic carrier [40], is a good candidate for the purpose. On the one hand, liposomes modified with polyethylene glycol (PEG) can extend the drug’s biological half-life [41] and thus reduce the dosing frequency. On the other hand, nanosized liposomal drugs can benefit from the enhanced permeability and retention effect [42] and active targeting approaches to accumulate in disease sites more precisely [43]. The loaded drug can be released intendedly by environmental cues with delicate lipid designs [44].

To achieve the therapeutic efficacy, loading an adequate drug amount into liposomes is of paramount importance. Remote (or active) loading is among the most efficient method for amphipathic drug loading, yielding the encapsulation efficiency much higher than the passive loading method [45]. Remote loading typically requires a trapping agent entrapped in the aqueous compartments of liposomes and proceeds in a proper extraliposomal medium, wherein a transmembrane chemical gradient formed by the trapping agent facilitates drug transport and accumulation [46,47,48]. A number of drug-trapping agent pairs have been reported for liposome drug loading, for example, calcium acetate for loading amphipathic weak-acid drug nalidixic acid [47] and ammonium sulfate for loading amphipathic weak-base drugs such as doxorubicin [46] and irinotecan [49]. However, not all the amphipathic drugs can be loaded in a remote loading fashion, because a drug must have appropriate properties, such as the solubility, lipophilicity, and dissociation constant of the ionizable group, to be efficiently loaded [50,51].

**Figure 1:**
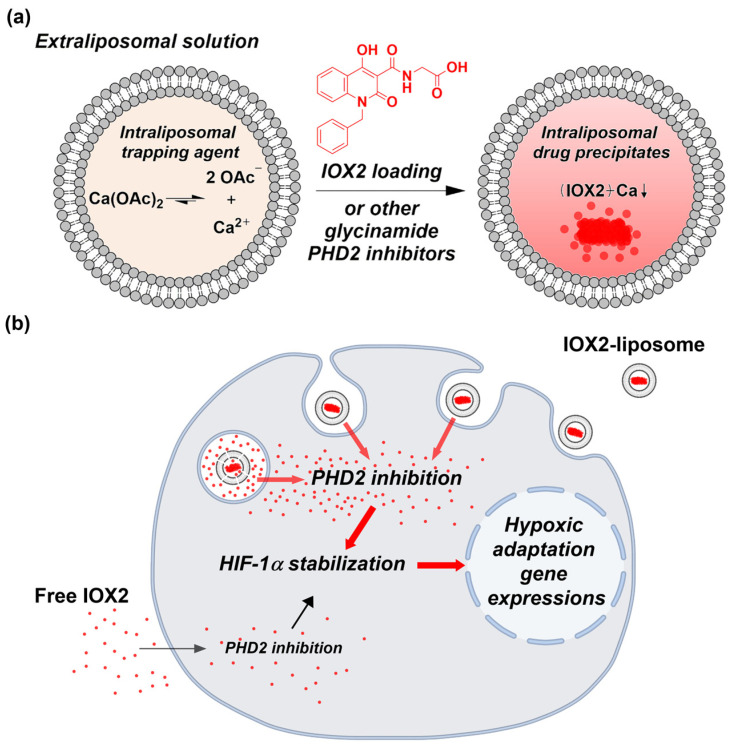
A schematic illustration of liposomal prolyl hydroxylase domain-containing protein 2 (PHD2) inhibitors and its intracellular delivery for inducing hypoxic adaptation responses. (**a**) Remote loading of IOX2 or other glycinamide PHD2 inhibitors into the liposome via a transmembrane calcium acetate gradient. (**b**) Liposomal IOX2 outperforming free IOX2 in stabilizing the hypoxia-inducible factor (HIF)-1α protein levels through the PHD2 inhibition and thereby hypoxic adaptation gene expressions.

PHD2 inhibitors are typically amphipathic weak acids with shared pharmacophore features (Figure S1), essential for the drug efficacy [16,52,53,54], including the following: (1) a bidentate coordination group that binds to the ferrous center of PHD2; (2) a glycinamide group (or the isostere) that competes for α-KG by interacting with Arg-383 of PHD2; and (3) a heterocyclic scaffold that provides additional interactions. IOX2, a potent quinoline glycinamide PHD2 inhibitor with IC_50_ of 22 nM, has been demonstrated to induce HIF-1α levels [53,54] and improve the wound-healing activity of human skin keratinocytes and fibroblasts exposed to sulfur mustard [55]. In the present study, we described an efficient method by using calcium acetate as the trapping agent for remotely loading IOX2 into liposomes (Figure 1a), with a lipid formulation similar to a currently FDA-approved liposomal drug. More importantly, the liposome-encapsulated IOX2, particularly when combining a targeting ligand, is superior to free IOX2 in stabilizing HIF-1α protein levels in cells (Figure 1b). Furthermore, we demonstrated that this loading method works well for the other two clinically used inhibitors—vadadustat and roxadustat, suggesting that PHD2 inhibitors with similar features could be remotely loaded into liposomes by the same approach.

## 2. Materials and Methods

### 2.1. Materials

General chemicals and solvents were purchased from Sigma-Aldrich (St. Louis, MO, USA) and J.T. Baker (Radnor, PA, USA). 1,2-Distearoyl-*sn*-glycero-3-phosphatidylcholine (DSPC), 1,2-distearoyl-*sn*-glycero-3-phosphatidylethanolamine-N-[methoxy(polyethylene glycol)-2000] (DSPE-PEG_2000_), and 1,2-distearoyl-sn-glycero-3-phosphoethanolamine-N-[folate(polyethylene glycol)-2000] (DSPE-PEG_2000_-folate) were purchased from Avanti Polar Lipids (Alabaster, AL, USA). IOX2 and 4-(2-hydroxyethyl)-1-piperazineethanesulfonic acid (HEPES) were from Sigma-Aldrich. Calcium (II) acetate (Ca(OAc)_2_) and ethylenediaminetetraacetic acid (EDTA) were from J.T. Baker (Radnor, PA, USA). Dimethyloxallyl glycine (DMOG) and vadadustat were from AbMole BioScience (Houston, TX, USA). Roxadustat was from MedChemExpress (Monmouth Junction, NJ, USA). All three PHD2 inhibitors were dissolved in dimethyl sulfoxide (DMSO) as stock solutions and diluted for use. KB and HeLa cell lines were obtained from the American Type Culture Collection (Manassas, VA, USA).

### 2.2. Preparation of Liposomes with Transmembrane Calcium Acetate Gradients

Lipids consisting of DSPC, cholesterol, and DSPE-PEG_2000_ at a molar ratio of 45:50:5 were used for liposome preparation unless otherwise specified. To a flask, lipids dissolving in chloroform were mixed and dried at 50 °C to form a thin film with a rotatory evaporator. The dried lipid film was kept under a high vacuum overnight for the complete removal of the solvent. The obtained lipid film was hydrated with a 100 mM Ca(OAc)_2_ solution at 60 °C and then subjected to successive freeze-and-thaw cycles. The liposome suspension was extruded through a polycarbonate membrane with a 0.1 μm pore size at 65 °C with a Mini Extruder (Avanti Polar Lipids, Alabaster, AL, USA), forming homogenous unilamellar vesicles. A transmembrane calcium acetate gradient was then generated by exchanging the extralipsomal phase with a calcium acetate-free solution (as listed in Table 1), by size-exclusion chromatography with a Sepharose CL-4B-packed column (GE Healthcare, Pittsburgh, PA, USA). The phospholipid concentration was colorimetrically determined by the Bartlett assay.

### 2.3. Remote Loading of PHD2 Inhibitors into Liposomes

All drug loading experiments were carried out by co-incubating the as-prepared Ca(OAc)_2_-liposomes and a certain amount of drugs (in terms of the drug-to-lipid molar ratio) at 65 °C, which was close to the transition temperature of DSPC for more efficient cross-membrane transport. Practically, an aliquot of 25 μL drug solution was added to 475 μL of liposome suspension to achieve the final lipid concentration of 5 mM and the desired drug-to-lipid molar ratio. A remote loading was initiated at a 65 °C water bath and typically lasted 30 min. For loading kinetics studies, IOX2 was mixed with the preformed liposomes at a drug-to-lipid molar ratio of 0.1 and incubated for the indicated time. At each time point, the loading process was stopped by moving the suspension onto ice for a few minutes and then the ambient. Unencapsulated drugs were then removed from the liposomes using a CL-4B-packed size-exclusion column pre-equilibrated with HEPES buffer solution (145 mM NaCl, 17 mM HPES, pH 7.4), yielding purified drug-loaded liposomes.

### 2.4. Quantification of Liposome-Encapsulated Drugs

Liposome-encapsulated PHD2 inhibitors were quantified by high-performance liquid chromatography (HPLC) analysis (Agilent Technologies, Santa Clara, CA, USA). Drug-loaded liposomes were ruptured for full drug release by adding 50% (*v/v*) ethanol followed by ultrasonication by a bath-type sonicator for 30 min. The liposome samples were then injected onto a reverse-phase column (Discovery BIO Wide Pore C5, 25 cm × 4.6 mm, 5 μm, Supelco, Bellefonte, PA, USA) and analyzed using a gradient elution, starting at 70% mobile phase A and 30% mobile phase B and linearly ramping up to 100% B in 20 min at a flow rate of 1.0 mL/min. Mobile phase A consisted of 5% (*v/v*) acetonitrile and 0.1% (*v/v*) trifluoroacetic acid in water, and mobile phase B consisted of 95% (*v/v*) acetonitrile and 0.1% (*v/v*) trifluoroacetic acid in water. The chromatographic peaks of IOX2—vadadustat, and roxadustat—were monitored by their absorption maxima (331 nm, 318 nm, and 348 nm, respectively), and the drug quantities were inferred by interpolating with established the standard curve of each drug using the peak areas. Drug loading efficiency or encapsulation efficiency was expressed as the percentage of drug-to-lipid molar ratio after loading over the drug-to-lipid molar ratio initially added:

(1) $$\mathrm{Encapsulation}\;\mathrm{efficiency}\left(\%\right)=\frac{\mathrm{Drug-to-lipid}\;\mathrm{molar}\;\mathrm{ratio}\;\mathrm{after}\;\mathrm{loading}}{\mathrm{Initial}\;\mathrm{drug-to-lipid}\;\mathrm{molar}\;\mathrm{ratio}}\times 100\%$$

### 2.5. Cryo-EM Imaging of Drug-Loaded Liposomes

The liposome structures were examined by the FEI Tecnai G2 F20 TWIN TEM (FEI, Hillsboro, OR, USA). A 200-mesh copper grid-supported holey carbon film (HC200-Cu, Electron Microscopy Sciences, Hatfield, PA, USA) was glow-discharged in an argon/oxygen atmosphere for 15 s. Four microliters of the liposome sample containing ~0.5 mM lipids were pipetted onto the copper grid, paper-blotted for 3 s in a 100% humidified chamber at 4 °C and plunge-frozen into liquid ethane cooled by liquid nitrogen using a Vitrobot system (FEI, Hillsboro, OR, USA). The grids were stored in liquid nitrogen until mounted for imaging. EM imaging was conducted in bright-field mode at an operating voltage of 200 kV. Images were recorded at a defocus value of ~1.8 μm under low-dose exposures (25–30 e/Å^2^) with a 4k × 4k charge-coupled device camera (Glatan, Pleasanton, CA, USA) at a magnification of 50,000×. All experiments were carried out at the Academia Sinica Cryo-EM Facility (Taipei, Taiwan).

### 2.6. Preparation and Characterization of IOX2-Loaded Liposomes for Cell Experiments

Lipids consisting of DSPC/cholesterol/DSPE-PEG_2000_ (molar ratio: 45:50:5) and DSPC/cholesterol/DSPE-PEG_2000_/DSPE-PEG_2000_-folate (molar ratio: 45:50:4:1) were used to generate Ca(OAc)_2_ liposomes. IOX2-loaded liposomes were generated by co-incubating IOX2 and the preformed Ca(OAc)_2_-liposome at a drug-to-lipid molar ratio of 0.12 at 65 °C for 30 min and further purified with a CL-4B-packed size-exclusion column pre-equilibrated with a HEPES buffer solution (145 mM NaCl, 17 mM HPES, pH 7.4). Alongside the IOX2-liposomes, IOX-free (empty) liposomes were prepared by a blank loading without IOX2, serving as a negative control group for the drug efficacy assay. The resultant liposome suspensions were ~20× concentrated using a centrifugal ultrafiltration device with a 100 kDa molecular weight cutoff (Vivaspin^®^6, Sartorius, Göttingen, Germany) at 2000–3000× *g*, with no IOX2 leakage in the filtrates monitored by UV-visible spectrometry. The concentrated liposomes were taken for lipid and IOX2 quantification as described above. Typically, our preparation generated liposome suspensions to the final lipid concentration around 30 mM. The size and ζ-potential of the liposomes were measured by a Zetasizer Ultra (Malvern Panalytical, Malvern, Worcestershire, UK). The liposome samples were diluted with a HEPES buffer solution to a final lipid concentration of 0.05 mM for nanoparticle analysis. All measurements were conducted at 25 °C.

### 2.7. Liposomal IOX2 Treatment in Cell Experiments

Human cervical carcinoma KB cells were routinely cultured at 37 °C in 5% CO_2_/95% air in a humidified incubator using folic acid-free Roswell Park Memorial Institute (RPMI) 1640 medium (Gibco, Waltham, MA, USA) supplemented with 10% heat-inactivated fetal bovine serum (Biological Industries, Beit HaEmek, Israel), 100 unit/mL penicillin, and 100 μg/mL streptomycin (Gibco, Waltham, MA, USA). For drug efficacy assays, 3 × 10^5^ KB cells were seeded on each well of a 6-well culture plate and grown in an RPMI 1640 medium. When reaching a ~90% confluency, the cells were washed once with warm isotonic phosphate-buffered saline (PBS) and then separately treated with IOX2, IOX2-liposomes, and 1% folate-decorated IOX2-liposomes at final IOX2 concentrations of 5 mM, 10 mM, 20 mM, 50 mM, and 100 mM in the RPMI 1640 medium for 6 h. A membrane-permeable α-KG-dioxygenase inhibitor DMOG was used as a positive control, and DMSO and the empty liposomes were used as vehicle controls for free IOX2 and IOX2-liposomes, respectively. The cells were also co-treated with 0.5 mM folic acid and 1% folate-decorated liposomes for targeting ligand competition. HeLa cells were treated with IOX2 and IOX2-liposomes following the same procedure, but using a Dulbecco’s modified eagle medium (Gibco, Waltham, MA, USA) instead of an RPMI 1640 medium.

### 2.8. Liposomal IOX2-Induced HIF-1α Stabilization Evaluated by Western Blotting

In the evaluation of HIF-1α protein expression as an indicator of PHD2 inhibition, the treated cells were washed twice with ice-cold PBS and lysed in a RIPA buffer (150 mM NaCl, 1%, NP-40, 1% sodium deoxycholate, 0.1% sodium dodecyl sulphate (SDS), and 25 mM Tris, pH 7.6) with protease inhibitors (Calbiochem^®^, San Diego, CA, USA). Cell extracts were ultrasonicated for 1 min using a bath-type sonicator and then centrifuged at 14,000× *g* for 15 min at 4 °C. After removing the pellets, 30 μg of protein extracts, as determined by BCA protein assay, were electrophoresed on 8% SDS-polyacrylamide gels and transferred onto PVDF membranes (PerkinElmer, Waltham, MA, USA) for Western blotting. The membranes were blocked with 3% (*w/v*) bovine serum albumin (BSA) in a TBST buffer (140 mM NaCl, 3 mM KCl, 0.1% Tween-20, and 25 mM Tris, pH 7.4) at room temperature for 1 h and then probed with an anti-HIF-1α mouse monoclonal antibody (1:1000; Clone 54/610958; BD Transduction Laboratories, San Diego, CA, USA), anti-β-actin mouse monoclonal antibody (1:5000; sc-47778; Santa Cruz Biotechnology, Dallas, TX, USA), or an anti-PHD2 rabbit polyclonal antibody (1:1000; NB100-137; Novus Biologicals, littleton, CO, USA) overnight at 4 °C. The membranes were washed four times with TBST and then incubated with horseradish peroxidase (HRP)-conjugated mouse IgGκ-binding protein (1:5000; sc-516102; Santa Cruz Biotechnology, Dallas, TX, USA) or mouse anti-rabbit IgG polyclonal antibody (1:5000; sc-2357; Santa Cruz Biotechnology, Dallas, TX, USA) in TBST with 3% BSA at room temperature for 1 h. After four-time washing with TBST, the chemiluminescent signals of the bound HRPs on the membranes were detected with a SuperSignal chemiluminescent substrate (Thermo Scientific, Waltham, MA, USA) in an iBright Imaging System (Invitrogen, Waltham, MA, USA). Two gels were electrophoresed, transferred and blotted in parallel with the same conditions to reduce run-to-run variations. A DMOG-positive control lysate was applied on the two gels to confirm generating the same blotting results (see Section 3.3).

## 3. Results and Discussion

### 3.1. IOX2 Loading into Liposomes via a Transmembrane Calcium Acetate Gradient and the Optimization for the Encapsulation Efficiency

In a remote loading, a drug must have at least one weakly ionizable function group in response to a transmembrane pH imbalance and sufficient lipophilicity to permeate the lipid bilayer. IOX2 is a quinoline glycinamide derivative with a carboxylic acid group (pKa = 3.35) (Figure 1a). Given the calculated logD values of IOX2 distribute from an acidic pH value of ~0.9 to at alkaline pH value of ~–5 (Figure S2), indicating pH-dictated lipophilicity, a built-in cross-membrane calcium acetate gradient leading to a pH imbalance (higher pH inside) [47] would facilitate its liposome loading. In another aspect, a metal ion can interact with a drug to form a coordination complex and contribute to the drug uptake and retention [56,57]. Based on the protein crystallography results [53,54], IOX2 is plausible to coordinate with the ferrous ion of PHD2 by the amide carbonyl and the adjacent hydroxyl group. Therefore, IOX2 may interact with the entrapped calcium ions to facilitate its accumulation in liposomes.

The liposomes with built-in calcium acetate gradients (Ca(OAc)_2_-liposome) were used for loading IOX2, and the encapsulation efficiency was optimized by a series of extraliposomal solutions as listed in Table 1. In the case of 10 mM NaCl, the encapsulation efficiency was only 43%, and prolonging incubation to 60 min only slightly improved the efficacy to ~46%. It is noteworthy that the encapsulation efficiency appreciably increased to 84.8% when using a 10 mM HEPES buffer (pH 7.4) as the extraliposomal solution. This improvement could be that the aqueous solubility of IOX2 was also significantly increased at pH 7.4 in the HEPES buffer (877 ± 101 μg/mL) in contrast to that in water (15.1 ± 2.3 μg/mL) (Figure S3). Nevertheless, this adjustment did not result in a complete IOX2 loading in the setting. It is likely that the residual calcium ions that remained in the exchanged extraliposomal solution interacted with IOX2, thereby interfering with the loading process. To completely remove the extraliposomal calcium ions, the chelating agent EDTA was included in the extraliposomal solution. As expected, the encapsulation efficiency became nearly complete (96.8%) in the 10 mM HEPES buffer (pH 7.4) with 1 mM EDTA. The high encapsulation efficiency was not affected when including 150 mM NaCl in the extraliposomal solution to balance the tonicity. To confirm that IOX2 was encapsulated in the liposome interior rather than associated with the membrane, a gradient-free liposome entrapped 150 mM NaCl solution was tested and resulted in only a 1.8% loading, indicating negligible IOX2–membrane interactions involved.

**Figure 2:**
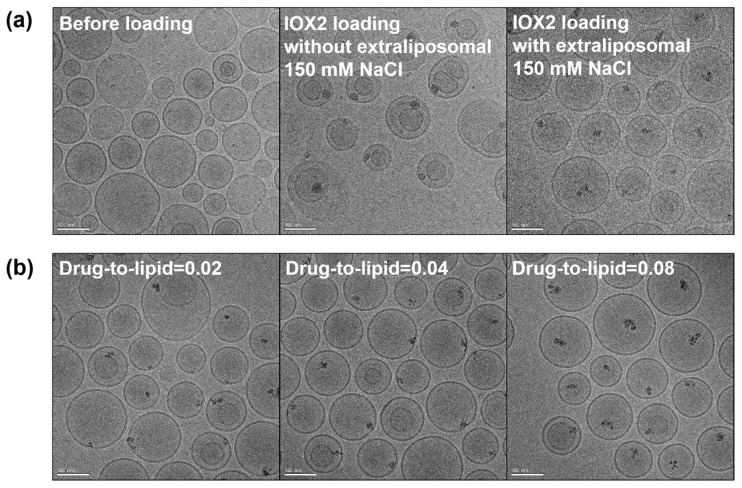
(**a**) Cryo-EM images of Ca(OAc)_2_-liposomes and IOX2-liposomes before and after loaded with IOX2 in 10 mM HEPES buffers (pH 7.4) with and without 150 mM NaCl. (**b**) Dose-dependent Ca-IOX2 precipitate formation in the IOX2-liposomes at different drug-to-lipid molar ratios. The scale bars represent 100 nm.

The physical state of an encapsulated drug inside liposomes is closely related to the trapping agent and can affect the drug’s retention and pharmacokinetics [49]. A sufficient payload must be stably encapsulated in liposomes for achieving a therapeutically effective dose. In this regard, the formation of an intraliposomal drug precipitate that keeps the drug gradient (lower concentration inside) during the loading and decouples from the acid-base equilibrium across the membrane is favorable [58]. In supporting that calcium acetate is a suitable trapping agent for precipitating the loaded IOX2 in the liposomes, rapid IOX2 precipitation was demonstrated after adding soluble IOX2 to a solution of 100 mM calcium acetate (pH 7.34), and the IOX2 solubility was found to be over 11-fold lower than that in the HEPES buffer (Figure S3). To see whether IOX2 can undergo intraliposomal nanoprecipitation by the Ca(OAc)_2_-mediated remote loading, IOX2-loaded liposomes were microscopically imaged by Cryo-EM. As shown in Figure 2, a single, electron-dense, and amorphous solid was found inside the most liposome interiors, together with the fact that the size of drug precipitates positively correlated to the amount of encapsulated IOX2 (Figure 2b and Figure S4), indicating the formation of Ca^2+^-IOX2 complexes. To our surprise, a large population of the liposomes had membrane structure changed from unilamellar to bilamellar, accompanied by drug deposits between two bilayers, after IOX2 loading with 10 mM HEPES (pH 7.4) plus 1 mM EDTA as the extraliposomal solution. Preforming a drug loading with a more isotonic HEPES-buffered saline (150 mM NaCl, 10 mM HEPES, and 1 mM EDTA, pH 7.4) was expected to circumvent this situation. As shown in Figure 2a, when using the isotonic HEPES-buffered saline, the unilamellar structure of the liposomes was preserved, while the size of intraliposomal drug precipitates remained unaffected.

Altogether, these results suggested that IOX2 can be stably encapsulated as nanoprecipitates in liposomes by remote loading via a transmembrane calcium acetate gradient. In line with previous findings that the enhanced solubility of other amphipathic compounds leads to successful remote loading [59,60], we found that in the extraliposomal solution at a buffered pH of 7.4, where IOX2′s aqueous solubility was superior to that of the unbuffered saline, IOX2 encapsulation was significantly improved. Using a metal chelator such as EDTA for removing extraliposomal calcium ions further increased the encapsulation to near 100% efficiency. A previously established model [50] has suggested that a remote-loadable amphipathic weak acid should have a pKa value of >3 and a log*D* value between −2.5 and 2 at pH 7. Although IOX2 seemed not an eligible candidate due to its off-range value (logD of ~−3.8 at pH 7; Figure S2), with the above-described optimization, IOX2 can be remote loadable in the high encapsulation efficiency.

### 3.2. Liposome Loading Kinetics and Capacity of IOX2 via a Calcium Acetate Gradient

**Figure 3:**
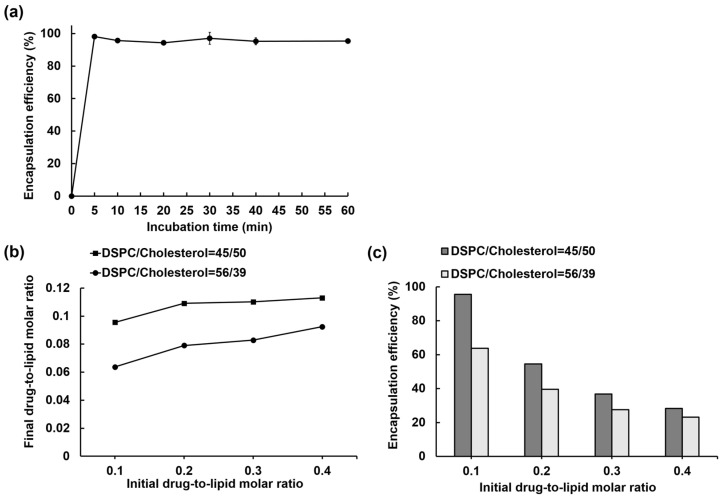
(**a**) Loading kinetics of IOX2 into liposomes with an optimal extraliposomal solution at a drug-to-lipid molar ratio of 0.1. (**b**) Dose-dependent IOX2 loading capacities of the liposomes containing 50% and 39% cholesterol. (**c**) Encapsulation efficiencies of the liposomes containing 50% and 39% cholesterol.

Rapid liposome drug loading relies on the adequate membrane permeability for the drug and the transmembrane gradient magnitude of a trapping agent. The loading kinetics of IOX2 into Ca(OAc)_2_-liposomes was investigated using an optimal extraliposomal solution (HEPES buffered saline with 1 mM EDTA) to estimate the loading rate. Drug encapsulation was nearly complete (98%) at the first time point (5 min) and remained constant (>95%) throughout the entire time course, suggesting that IOX2 can be rapidly loaded in the Ca(OAc)_2_-liposomes (Figure 3a).

To estimate the IOX2 loading capacity limit of the Ca(OAc)_2_-liposomes, the added IOX2 amount was gradually increased from 0.1 to 0.4 at the initial drug-to-lipid molar ratio, and the incubation time was 30 min to ensure loading saturation. The amount of IOX2 loaded into the liposomes (expressed as the final drug-to-lipid molar ratio) was plotted against the initially added drug-to-lipid molar ratio, as shown in Figure 3b. The final drug-to-lipid molar ratio was near 0.1 at an initial drug-to-lipid molar ratio of 0.1, and it increased to ~0.11 and plateaued when the initial drug ratios were increased to 0.2 and higher. Accordingly, the encapsulation efficiency was 55% at an initial drug-to-lipid molar ratio of 0.2 and fell to 37% and 28% at drug-to-lipid molar ratios of 0.3 and 0.4, respectively (Figure 3c). The results indicated that the Ca(OAc)2-liposomes had an IOX2 loading capacity limit of 0.11 in the final drug-to-lipid molar ratio.

The cholesterol content in a liposome composition can affect the drug loading capacity of the liposome [61,62], by altering the fluidity and phase behaviors of the lipid bilayer [63,64]. A lowered-cholesterol-content (39%) liposome, which was used in lipid compositions of Doxil^®^ and Onivyde^®^, was compared with a higher-cholesterol-content (50%) liposome to understand the effects of cholesterol on the IOX2 loading capacity. As shown in Figure 3b, the loading amounts of the 39%-cholesterol liposomes were significantly lower at all the initial drug-to-lipid molar ratios used in contrast to the 50%-cholesterol liposomes. As a result, the encapsulation efficiencies of the 50%-cholesterol liposomes were higher than those of the 39%-cholesterol liposomes (Figure 3c). The results suggested that cholesterol can impact the IOX2 loading capacity of the Ca(OAc)_2_-liposomes, in agreement with the previous finding that cholesterol in liposomes is essential for high idarubicin encapsulation [61], as it maintains the interior pH with a lower proton permeability of the membrane that is required for stable drug loading [62].

### 3.3. Comparison of Free IOX2 and Liposomal IOX2-Induced HIF-1α Stabilization in Cells

The abilities to stabilize the HIF-1α of liposomal IOX2 and free IOX2 were compared at the cellular level to evaluate if the liposome formulation could improve the drug efficacy. The folate-functionalized targeted IOX2-liposome was also evaluated to see if the drug efficacy could be boosted by the targeted delivery approach [65,66]. It is worth noting that the IOX2-liposomes can provide an apparent IOX2 concentration beyond its aqueous solubility limit (~2.5 mM at pH 7.4), highlighting another advantage of nanoformulation in pharmaceutical applications. The characterization results of the liposomes are summarized in Table S1. After the IOX2 loading, hydrodynamic diameters for the folate-free and 1% folate-decorated liposomes were ~110 nm and ~100 nm, respectively. The ζ-potentials of the folate-liposomes (~−3 mV) were slightly negative than those of folate-free liposomes (−1 mV to −2 mV), probably due to the negatively charged folates present on the liposome surface.

**Figure 4:**
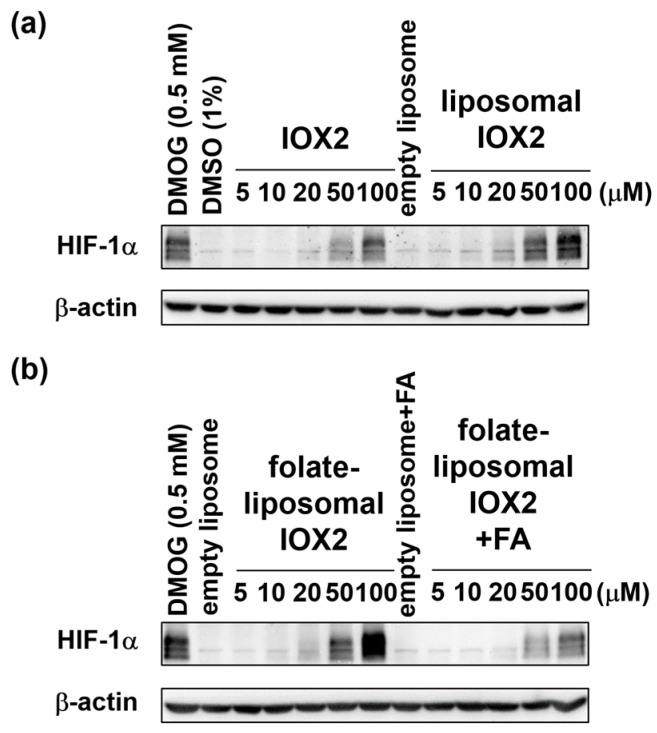
HIF-1α protein levels in KB cells stabilized by free IOX2 and liposomal IOX2 (**a**) and folate-liposomal IOX2 (**b**) in the presence and absence of excess folic acid (FA).

KB cells, normally overexpressing folate receptors [67], were treated with free IOX2, IOX2-liposomes, and folate-IOX2-liposomes at the same apparent IOX2 concentration ranging from 5 μM to 100 μM under normoxic conditions for 6 h. While all IOX2 treatment groups increased the HIF-1α levels compared to the respective control groups, liposomal IOX2 induced higher HIF-1α levels than those by free IOX2, especially at 50 μM and 100 μM (Figure 4a). More importantly, the targeted folate-liposomal IOX2 induced even higher HIF-1α levels when compared to the nontargeted liposomal IOX2, especially at 100 μM (Figure 4b). As expected, the co-treatment with excess folic acid that competed for the receptors diminished the effects (Figure 4b). HeLa cells were also given the same treatment and came to the same conclusion (Figure S5a), suggesting that the efficacy improvement was not cell-specific. It was also found that when prolonging the treatment time to 24 h, the intracellular HIF-1α decreased significantly (Figure S5b), which indicated the uptake and metabolizing peak of IOX2 occurred earlier than 24 h in the given experimental conditions.

Collectively, these results clearly showed that liposomal IOX2 had a higher drug efficiency than free IOX2 and suggested that the liposome-uptake pathway outperforms simple diffusion that mediates the entry of extracellular free IOX2 into cells (Figure 1b). Furthermore, this efficacy improvement would be further strengthened by targeting mechanisms, as demonstrated herein by folate-liposomal IOX2, which showed more potent than its nontargeted counterpart in KB cells.

### 3.4. Remote Loading of other PHD2 Inhibitors into Liposomes via a Calcium Acetate Gradient

**Figure 5:**
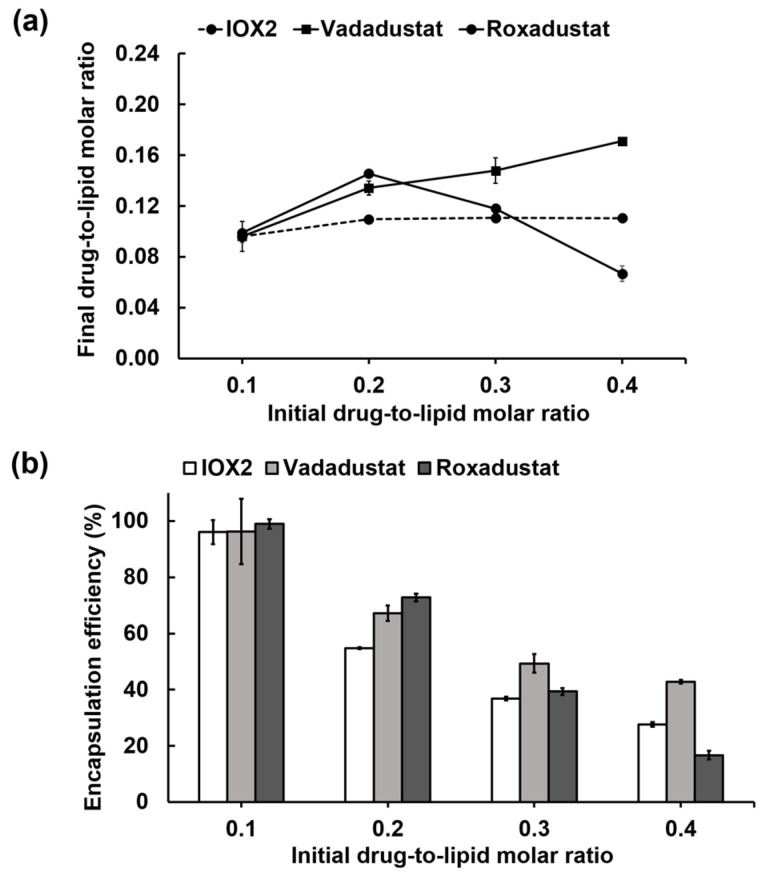
Remote loading of PHD2 inhibitors vadadustat, and roxadustat into liposomes in comparison with IOX2. (**a**) Dose-dependent drug loading capacities at different initial drug-to-lipid molar ratios. (**b**) Encapsulation efficiencies at different initial drug-to-lipid molar ratios.

Given the shared pharmacophore features, we anticipated that the developed loading method also works for other heterocyclic glycinamide PHD2 inhibitors. Two clinically used PHD2 inhibitors—vadadustat and roxadustat, representing pyridine- and isoquinoline-based glycinamide derivatives, respectively (Figure S1), were also tested for the remote loading. The Ca(OAc)_2_-liposomes in the composition of DSPC/cholesterol/DSPE-PEG_2000_ (molar ratio: 45:50:5) were used for loading these drugs in an optimal extraliposomal solution at 65 °C for 30 min. While vadadustat and roxadustat also showed a ~100% loading at an initial drug-to-lipid molar ratio of 0.1, both drugs showed higher encapsulation efficiencies than IOX2 when using an initial drug-to-lipid molar ratio of 0.2 (Figure 5). For Vadadustat, a final drug-to-lipid molar ratio of ~0.17 was obtained when using an initial drug-to-lipid molar ratio of 0.4 (Figure 5a). For Roxadustat, the loading amount peaked at a drug-to-lipid molar ratio of 0.2, resulting in a final drug-to-lipid molar ratio of 0.15, which achieved the usual encapsulation ratio of commercial liposomal drugs. Due to the intrinsically low solubility of Roxadustat, using higher initial drug-to-lipid molar ratios (0.3 and 0.4) did not increase the loading amount and the encapsulation efficiency (Figure 5). Encapsulation at higher drug-to-lipid molar ratios could be further explored by using other complicated means to overcome solubility limitation [68].

These results supported our hypothesis that PHD2 inhibitors with similar pharmacophore features could be remotely loaded into liposomes using the developed method. We envisioned that the liposome-formulated PHD2 inhibitor by the loading method could enhance the drug efficiency in stabilizing HIF-1α and hence hypoxia-adaptive responses while holding the promise of preferable biodistributions through targeting mechanisms. Accordingly, these PHD2 inhibitors would have therapeutic uses towards more precise, tissue-targeted drug actions (and thereby reduced systemic effects) for treating disease lesions that are highly localized [69], such as ischemia-reperfusion injury and solid tumors.

## 4. Conclusions

In this study, we reported an efficient method for remotely loading a quinoline-based glycinamide PHD2 inhibitor IOX2 into nanoliposomes. Driven by a transmembrane calcium acetate gradient, IOX2 was encapsulated as nanoprecipitates in unilamellar liposomes with a high encapsulation efficiency through the optimization of an extraliposomal solution. The liposome-encapsulated IOX2 exhibited a higher drug efficacy in stabilizing HIF-1α when compared with free IOX2, and the efficacy was further strengthened in conjunction with the folate-mediated targeting. This loading method could be generally applied for other heterocyclic glycinamide PHD2 inhibitors, as demonstrated here by clinically used inhibitors vadadustat and roxadustat. With liposome formulation, PHD2 inhibitors would be a potential therapeutic with reduced systemic toxicity for treating more specifically localized disease lesions such as ischemia-reperfusion injury and solid tumors.

## Figures and Tables

**Table 1:** Encapsulation efficiencies of IOX2 with remotely loaded liposomes under various loading conditions.

Intraliposomal Trapping Solution	Extraliposomal Solution	Encapsulation Efficiency (%)
150 mM NaCl	150 mM NaCl	1.8
100 mM Ca(OAc)_2_	10 mM NaCl	43.0
		46.4 *
	10 mM HEPES (pH 7.4)	84.8
	10 mM HEPES (pH 7.4) 1 mM EDTA	96.8
	150 mM NaCl 10 mM HEPES (pH 7.4) 1 mM EDTA	~100

* The loading duration was 60 min, instead of 30 min.

## Data Availability

The data presented in this study are available on request from the corresponding author.

## Supplementary Materials

The following are available online at https://www.mdpi.com/article/10.3390/nano12010163/s1, Figure S1. Examples of PHD2 inhibitors with similar pharmacophore features, Figure S2. LogD profile for IOX2 calculated by ChemAxon software, Figure S3. IOX2 solubility values in various media at room temperature (r.t.) and an elevated temperature (65 °C), Figure S4. Cryo-EM images of IOX2-liposomes showing the dose-dependent Ca-IOX2 precipitate formation in the IOX2-liposomes at drug-to-lipid molar ratios of (a) 0.02, (b) 0.04, and (c) 0.08. The scale bars represent 100 nm, Figure S5. HIF-1α protein levels in HeLa cells stabilized by free IOX2 and liposomal IOX2 at (a) 6 h and (b) 24 h. The time-dependent results showed the decreased drug effects in both treatment groups at 24 h, suggesting that the peak of IOX2 uptake and metabolism occurs sooner than 24 h. Table S1. Particle size and ζ-potential analyses of IOX2-liposomes.
