# Supplementary material for: Liposomal PHD2 Inhibitors and the Enhanced Efficacy in Stabilizing HIF-1α
Source: Nanomaterials (Basel). 2022 Jan 3;12(1):163. doi: 10.3390/nano12010163 (PMC8746909; doi:10.3390/nano12010163)
Supplement: Supplementary file 1 [file nanomaterials-12-00163-s001.zip › nanomaterials-1494759-supplementary.pdf]

nanomaterials

MDPI

*Article*

# Liposomal PHD2 Inhibitors and the Enhanced Efficacy in Stabilizing HIF-1α

**Cheng-Bang Jian [1,2,3], Xu-En Yu [1,4], Hua-De Gao [1,2], Huai-An Chen [1], Ren-Hua Jheng [1,4], Chong-Yan Chen [1] and Hsien-Ming Lee [1,*]**

1 Institute of Chemistry, Academia Sinica, Taipei 11529, Taiwan; r01223223@ntu.edu.tw (C.-B.J.); allenyu2932@gmail.com (X.-E.Y.); student719@msn.com (H.-D.G.); eam012331@gmail.com (H.-A.C.); renhuajheng@gmail.com (R.-H.J.); andy6111ha@gmail.com (C.-Y.C.)
2 Department of Chemistry, National Taiwan University, Taipei 10617, Taiwan
3 Nano Science and Technology Program, Taiwan International Graduate Program, Academia Sinica and National Taiwan University, Taipei 11529, Taiwan
4 Department of Chemistry, National Central University, Taoyuan City 320317, Taiwan
* Correspondence: leehm@chem.sinica.edu.tw; Tel.: +886-2-5572-8620

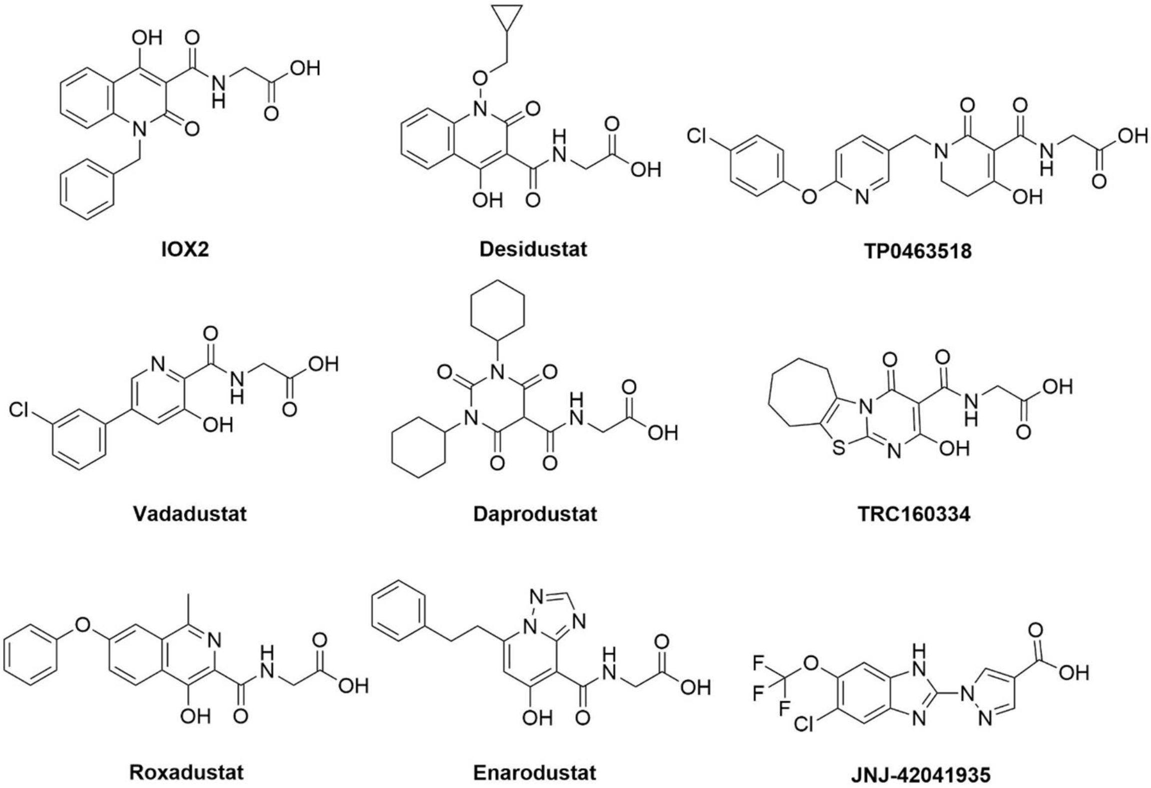


**Figure S1.** Examples of PHD2 inhibitors with similar pharmacophore features.

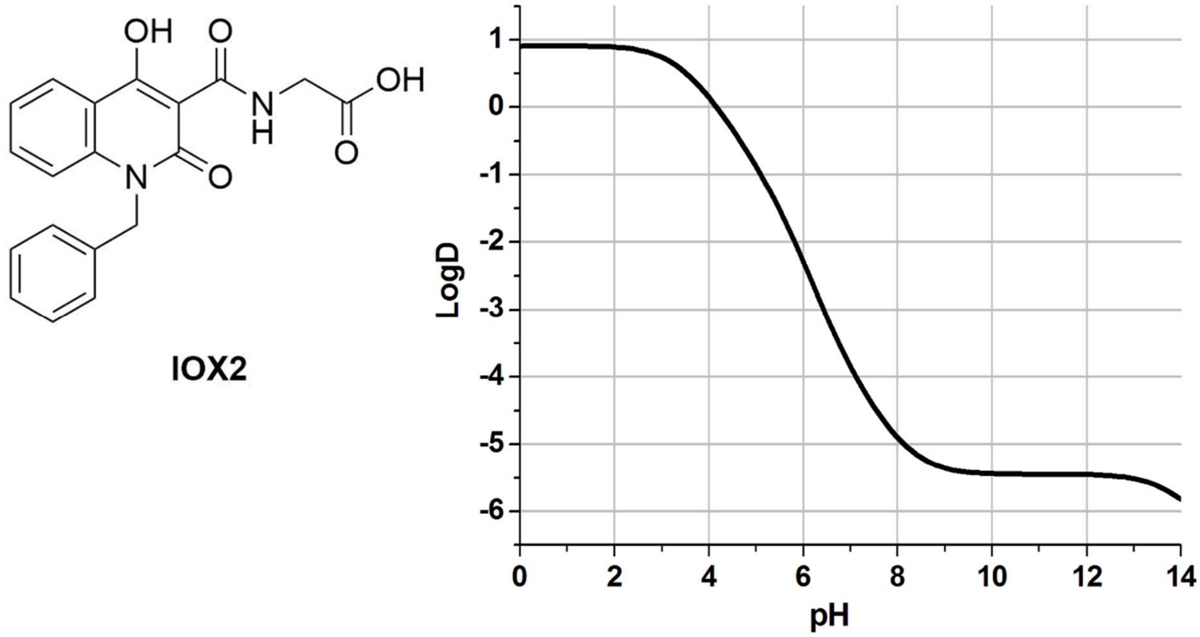


**Figure S2.** LogD profile for IOX2 calculated by ChemAxon software [1].

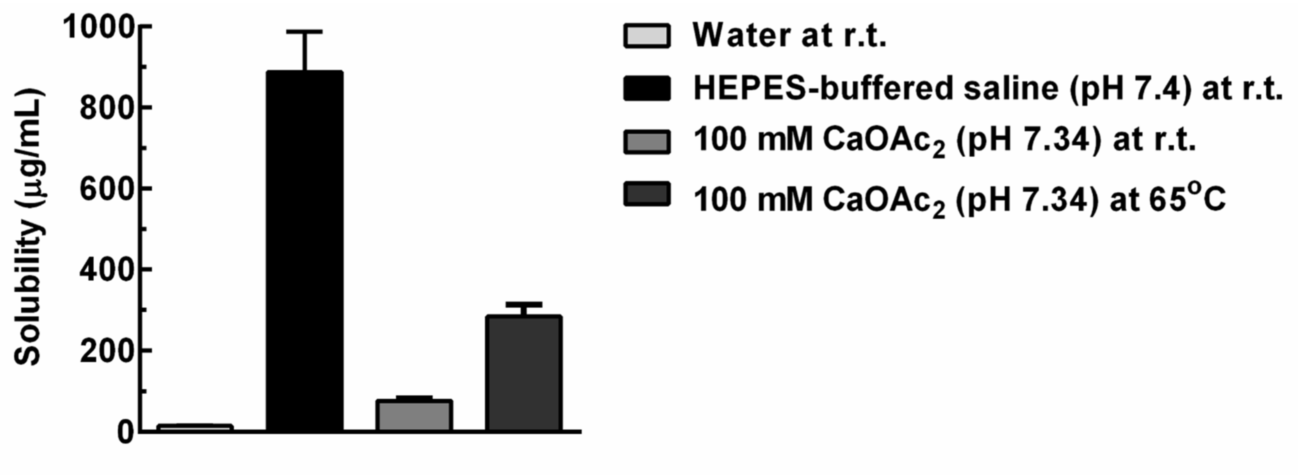


**Figure S3.** IOX2 solubility values in various media at room temperature (r.t.) and an elevated temperature (65 °C).

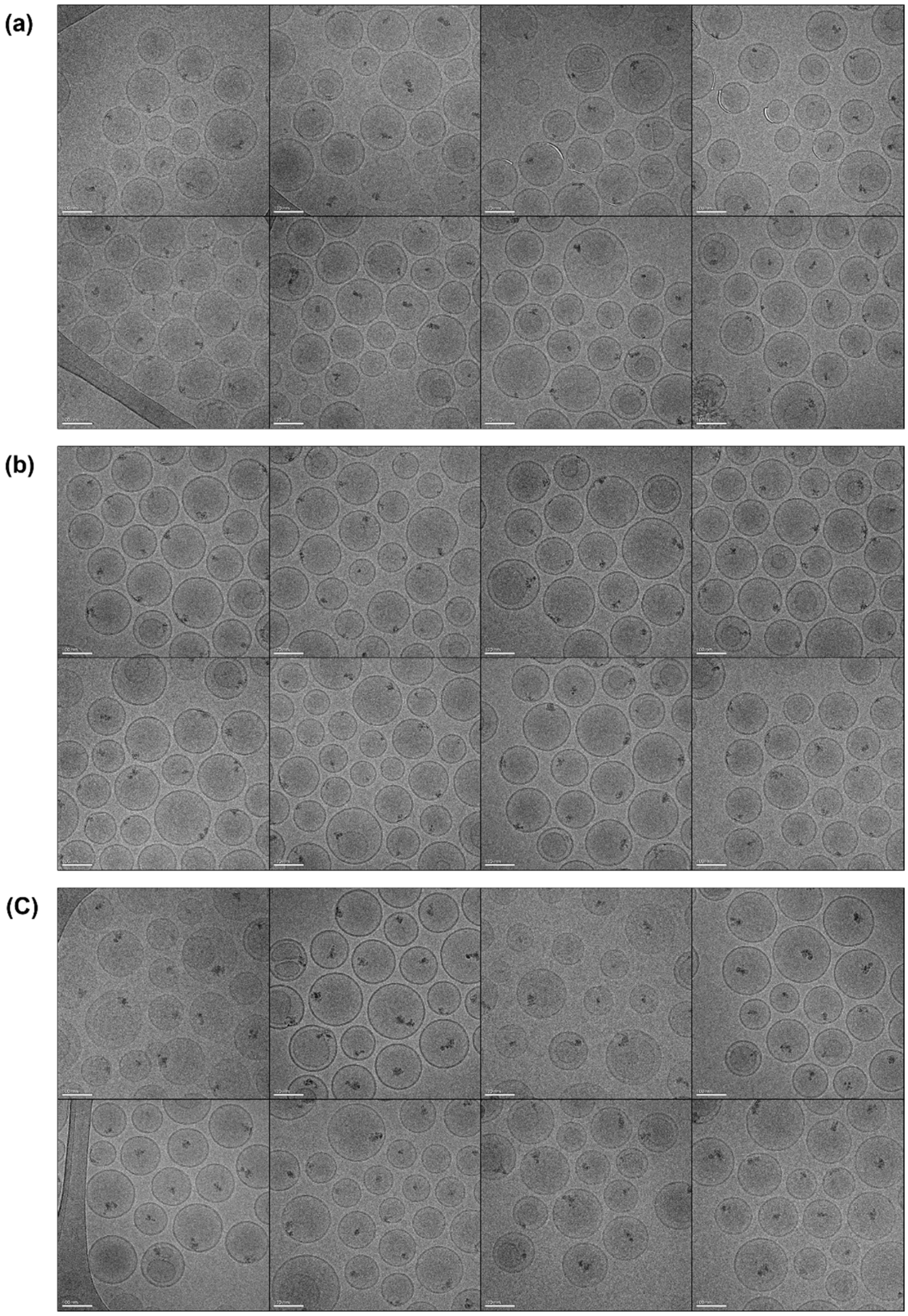


**Figure S4.** Cryo-EM images of IOX2-liposomes showing the dose-dependent Ca-IOX2 precipitate formation in the IOX2-liposomes at drug-to-lipid molar ratios of (**a**) 0.02, (**b**) 0.04, and (**c**) 0.08. The scale bars represent 100 nm.

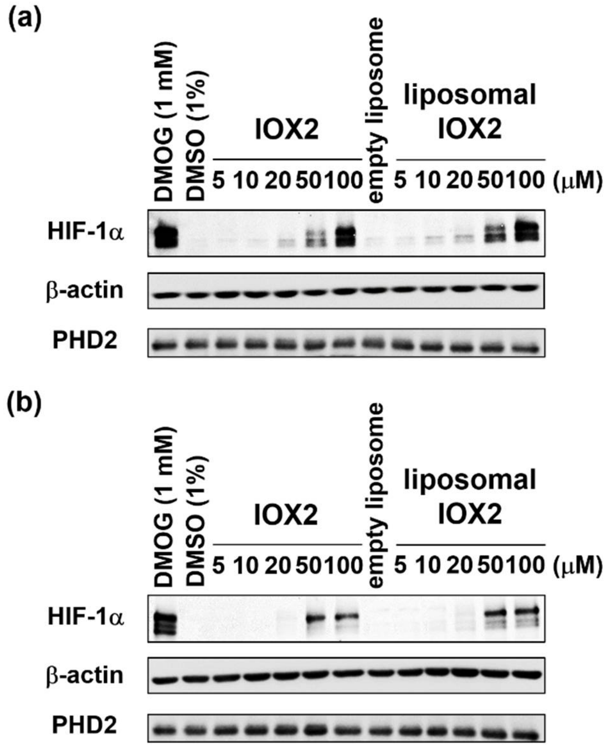


**Figure S5.** HIF-1α protein levels in HeLa cells stabilized by free IOX2 and liposomal IOX2 at (**a**) 6 h and (**b**) 24 h. The time-dependent results showed the decreased drug effects in both treatment groups at 24 h, suggesting that the peak of IOX2 uptake and metabolism occurs sooner than 24 h.

**Table S1.** Particle size and ζ-potential analyses of IOX2-liposomes.

| Liposome Composition | | Particle Size (nm) | PDI | ζ-Potential (mV) |
|---|---|---|---|---|
| DSPC/cholesterol/DSPE-PEG$_{2000}$=45:50:5 | Before loading | 116.4 ± 1.1 | 0.106 ± 0.028 | −1.53 ± 1.12 |
| | Blank loading | 110.9 ± 2.8 | 0.067 ± 0.027 | −1.08 ± 1.61 |
| | IOX2 loading | 109.3 ± 1.1 | 0.070 ± 0.011 | −2.02 ± 0.88 |
| DSPC/cholesterol/DSPE-PEG$_{2000}$/DSPE-PEG$_{2000}$-folate=45:50:4:1 | Before loading | 113.5 ± 2.2 | 0.188 ± 0.011 | −3.08 ± 0.87 |
| | Blank loading | 100.3 ± 1.7 | 0.075 ± 0.018 | −3.48 ± 1.50 |
| | IOX2 loading | 98.0 ± 0.7 | 0.072 ± 0.015 | −3.07 ± 0.74 |

## References

1. ChemAxon, Chemicalized - Instant Cheminformatics Solutions. 2021. Available online: https://chemicalize.com/welcome (accessed on 3 November 2021)
